# Expression of parathyroid hormone-related protein during immortalization of human peripheral blood mononuclear cells by HTLV-1: Implications for transformation

**DOI:** 10.1186/1742-4690-5-46

**Published:** 2008-06-09

**Authors:** Murali VP Nadella, Sherry T Shu, Wessel P Dirksen, Nanda K Thudi, Kiran S Nadella, Soledad A Fernandez, Michael D Lairmore, Patrick L Green, Thomas J Rosol

**Affiliations:** 1 Department of Veterinary Biosciences, The Ohio State University, Columbus, OH, USA; 2 Center for Retrovirus Research, The Ohio State University, Columbus, OH, USA; 3 Human Cancer Genetics, The Ohio State University, Columbus, OH, USA; 4 Center for Biostatistics, The Ohio State University, Columbus, OH, USA

## Abstract

**Background:**

Adult T-cell leukemia/lymphoma (ATLL) is initiated by infection with human T-lymphotropic virus type-1 (HTLV-1); however, additional host factors are also required for T-cell transformation and development of ATLL. The HTLV-1 Tax protein plays an important role in the transformation of T-cells although the exact mechanisms remain unclear. Parathyroid hormone-related protein (PTHrP) plays an important role in the pathogenesis of humoral hypercalcemia of malignancy (HHM) that occurs in the majority of ATLL patients. However, PTHrP is also up-regulated in HTLV-1-carriers and HTLV-1-associated myelopathy/tropical spastic paraparesis (HAM/TSP) patients without hypercalcemia, indicating that PTHrP is expressed before transformation of T-cells. The expression of PTHrP and the PTH/PTHrP receptor during immortalization or transformation of lymphocytes by HTLV-1 has not been investigated.

**Results:**

We report that PTHrP was up-regulated during immortalization of lymphocytes from peripheral blood mononuclear cells by HTLV-1 infection in long-term co-culture assays. There was preferential utilization of the PTHrP-P2 promoter in the immortalized cells compared to the HTLV-1-transformed MT-2 cells. PTHrP expression did not correlate temporally with expression of HTLV-1 tax. HTLV-1 infection up-regulated the PTHrP receptor (PTH1R) in lymphocytes indicating a potential autocrine role for PTHrP. Furthermore, co-transfection of HTLV-1 expression plasmids and PTHrP P2/P3-promoter luciferase reporter plasmids demonstrated that HTLV-1 up-regulated PTHrP expression only mildly, indicating that other cellular factors and/or events are required for the very high PTHrP expression observed in ATLL cells. We also report that macrophage inflammatory protein-1α (MIP-1α), a cellular gene known to play an important role in the pathogenesis of HHM in ATLL patients, was highly expressed during early HTLV-1 infection indicating that, unlike PTHrP, its expression was enhanced due to activation of lymphocytes by HTLV-1 infection.

**Conclusion:**

These data demonstrate that PTHrP and its receptor are up-regulated specifically during immortalization of T-lymphocytes by HTLV-1 infection and may facilitate the transformation process.

## Background

Human T-lymphotropic virus type I (HTLV-I) is the etiological agent of adult T-cell leukemia/lymphoma (ATLL), HTLV-1-associated myelopathy/tropical spastic paraparesis (HAM/TSP) and a variety of other disorders [1,2]. ATLL is an aggressive malignancy of CD4+ T cells that occurs in approximately 5% of infected individuals after a long latency period of 20–40 years. The long latency period and the relatively low proportion of HTLV-1-infected people that develop ATLL reflect the inefficiency of the virus to transform cells and the need for multiple cooperative changes in growth control mechanisms to induce leukemogenesis.

HTLV-1 is a complex deltaretrovirus and its genome not only encodes for the essential viral genes gag, pol, and env, but also additional HTLV-1-specific regulatory proteins Tax and Rex, several accessory proteins p12, p13, p30 and a minus-strand encoded protein, HTLV-1 bZIP-factor (HBZ) [7]. Although the precise mechanisms underlying transformation are not completely understood, the 40-kDa transcriptional transactivator, Tax, is thought to be principally responsible for tumorigenesis [8]. The ability to activate cellular genes, including proto-oncogenes, is a key mechanism leading to immortalization and transformation of HTLV-1-infected cells. Rex regulates the expression of incompletely spliced viral RNAs by interacting with the Rex response element in the viral RNA and cellular proteins used by CRM-dependent nuclear export [15]. Although Rex is not required for immortalization of lymphocytes *in vitro*, it is required for infectivity and persistence *in vivo *[16]. The accessory genes *p12*, *p30*, *p13 *and *HBZ *contribute to establishing persistent viral infection *in vivo *but are not required for transformation of cells *in vitro *[17,18].

About 80% of ATLL patients develop humoral hypercalcemia of malignancy (HHM), a life-threatening paraneoplastic syndrome that occurs in a wide variety of cancers in addition to ATLL [19]. ATLL cells express factors such as interleukin-1, tumor necrosis factor β, parathyroid hormone-related protein (PTHrP), macrophage inflammatory protein-1α (MIP-1α) and receptor activator of nuclear factor-κB ligand (RANKL) that directly and/or indirectly stimulate osteoclast differentiation and activity, resulting in hypercalcemia [20-24]. PTHrP has been shown to play a central role in the pathogenesis of HHM in ATLL patients, but likely has additive or synergistic effects with other tumor-associated cytokines [25]. Although PTHrP was discovered based on its role in the pathogenesis of HHM, PTHrP is now known to be a complex factor with a broad range of physiologic and/or pathophysiologic actions in different tissues [34]. PTHrP has been shown to be an auto/paracrine cell growth regulator that increases proliferation of several cell types including chondrocytes and renal epithelial cells [43]. PTHrP stimulates proliferation through the PTH1R by mechanisms involving both PKA and PKC signaling pathways.

Watanabe et al have shown that PTHrP was constitutively expressed in HTLV-1-carriers and ATLL patients with or without hypercalcemia which suggests that PTHrP is expressed before transformation of lymphocytes [26]. ATLL cell adhesion up-regulated PTHrP expression [27] indicating additional roles for PTHrP besides its central role in the pathogenesis of HHM. Moreover, PTHrP gene expression was induced during transformation of normal rat embryo fibroblasts by co-transfection with an activated *ras *gene and a mutated p53 gene [40]. Insogna et al have shown that PTHrP induced transformation of rat fibroblasts with epidermal growth factor [41]. In addition, co-transfection of rat embryonic fibroblasts with Tax and ras transformed the fibroblasts and they were highly tumorigenic *in vivo *[42]. Based on these findings, it is possible that PTHrP functions as a transforming factor in conjunction with other oncogenes.

The goal of this study was to investigate the expression of PTHrP, its receptor, and MIP-1α during the early stages of immortalization of human lymphocytes by HTLV-1. Using long-term liquid culture immortalization assays, we showed that PTHrP and PTH1R were markedly up-regulated during immortalization of T-lymphocytes. PTHrP expression did not correlate temporally with HTLV-1 tax expression and IL-2 stimulation. Co-transfection of HTLV-1 with a PTHrP P2/P3 luciferase reporter showed that PTHrP was up-regulated by HTLV-1 infection.

## Results

### HTLV-1-infected PBMCs proliferate beyond six weeks

**Figure 1:**
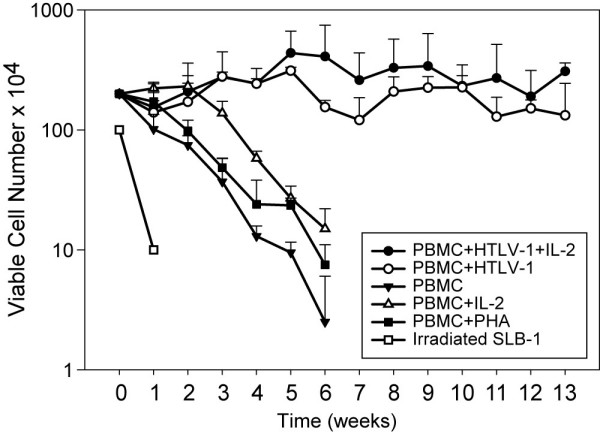
**Growth curves and p19 Gag expression in HTLV-1 T-lymphocyte immortalization assays**. Human PBMCs (2 × 10^6^) were cultured alone or with irradiated donor cells (SLB-1) in 24-well plates. Cell viability was measured weekly by trypan blue exclusion (0–13 weeks after co-cultivation) and growth curves are shown. PBMCs were infected with HTLV-1 in the presence of IL-2 (10 U/mL; supplemented from day 1 following HTLV-1 infection) or in the absence of IL-2. PBMCs with no stimulation, PBMCs stimulated with PHA and IL-2 irradiated SLB-1 cells served as controls. The results showed that only HTLV-1-infected cells continued to proliferate beyond 6 weeks in culture. Viable cell numbers were significantly different over time between treatment groups (p < 0.0001). While the PBMC+HTLV-1+IL-2 group cell numbers increased slightly over time, the remaining group cell numbers decreased over time, but the PBMC+HTLV-1 group cell numbers decreased only slightly. After using Dunnett's method to adjust for multiple comparisons, the HTLV-1-treated groups both had significantly higher cell numbers than the PBMC (control) group (p < 0.0001).

To investigate the expression of PTHrP early after HTLV-1 infection, we used long-term co-culture assays of PBMCs from healthy human donors with irradiated HTLV-1 producer cells (SLB-1) in the presence or absence of IL-2. Viable cells were counted by trypan blue exclusion and the results are shown in figure 1. Irradiated SLB-1 cells lived up to 1 week in culture. As expected, PBMCs grown in the absence of stimulation with either IL-2 or PHA, progressively decreased in numbers and failed to grow *in vitro *[31]. PBMCs supplemented with IL-2 or PHA lived and proliferated up to 2 weeks in culture, at which time they enter a "growth crisis" phase and decreased in numbers and lost viability beyond 6 weeks in culture. In contrast, HTLV-1-infected PBMCs continued to proliferate beyond 6 weeks for up to at least 13 weeks in culture. Cells that continued to proliferate beyond 8–9 weeks in culture in the presence or absence of exogenous IL-2 were referred to as immortalized cells. High levels of p19 Gag protein were detected throughout the co-culture demonstrating virus production (data not presented).

### PTHrP was up-regulated during immortalization of PBMCs with HTLV-1

**Figure 2:**
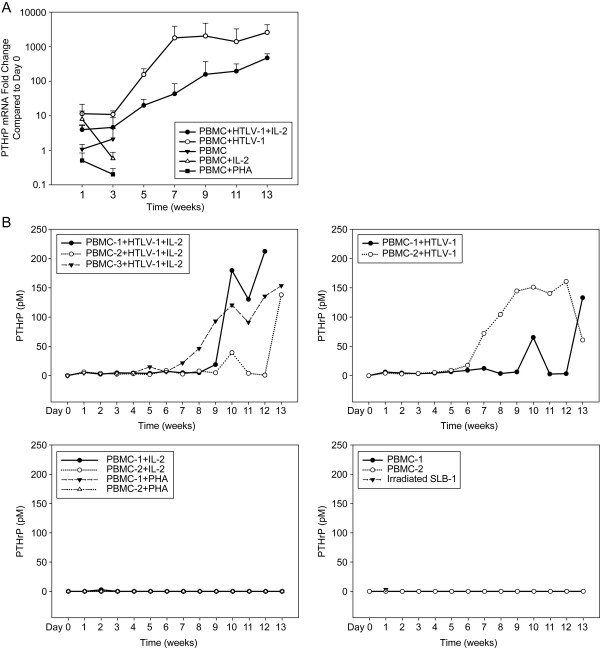
**PTHrP was markedly up-regulated during immortalization of PBMCs with HTLV-1 infection**. **(A) **PTHrP mRNA expression during immortalization of PBMCs with HTLV-1. Total RNA was extracted from the co-cultures at various time points and PTHrP mRNA expression was measured by real-time RT-PCR using the Taqman method. PTHrP expression was normalized to human β_2_M and the data were represented as fold change over uninfected PBMCs from day 0. After using Dunnett's method to adjust for multiple comparisons, the PBMC+HTLV-1 group was shown to have higher PTHrP mRNA level than the PBMC group (p < 0.0001). The PMBC+HTLV-1+IL-2 group was not different from the PBMC group due to the very limited data available for the PBMC group. These limited data were caused by low cell viability resulting in low RNA recovery from the PBMC group. **(B) **PTHrP protein expression during immortalization of PBMCs with HTLV-1. Secreted PTHrP was measured in the conditioned medium from the co-culture assays by IRMA. Results showed marked up-regulation of PTHrP secretion in PBMCs infected with HTLV-1 during the immortalization phase. PTHrP concentrations were significantly different over time between treatment groups (p < 0.0001). While PTHrP secretion increased in HTLV-1-treated groups over time, PTHrP secretion in the other 4 groups remained negligible and unchanged. After using Dunnett's method to adjust for multiple comparisons, both HTLV-1-treated groups had significantly higher protein levels than the PBMC group (p < 0.0001).

To determine the temporal expression of PTHrP during HTLV-1 immortalization of PBMCs, PTHrP mRNA (Figure 2A) and protein (Figure 2B) expression were analyzed at various time points during the long-term co-culture assays. Freshly-isolated PBMCs expressed very little PTHrP mRNA, which was barely detectable by RT-PCR. There was no increase in PTHrP mRNA or protein expression in unstimulated PBMCs during culture *in vitro*. IL-2 stimulation up-regulated PTHrP mRNA expression in the first week (3.8 to 12-fold) compared to unstimulated PBMCs. After one week, there was no further up-regulation of PTHrP mRNA in the IL-2-stimulated PBMCs. Although there was an increase in the PTHrP mRNA expression due to IL-2 stimulation, PTHrP protein (2.6 pM) was detectable in only one of the samples (PBMC-1 + IL-2). No increase in PTHrP mRNA or protein occurred with PHA stimulation of PBMCs. In contrast, HTLV-1 infection markedly up-regulated PTHrP mRNA expression compared to uninfected PBMCs. In PBMCs infected with HTLV-1 in the presence of IL-2, PTHrP mRNA was up-regulated 300- to 500-fold 5–11 weeks post co-culture compared to uninfected PBMCs at day 0. In PBMCs infected with HTLV-1 in the absence of IL-2, PTHrP mRNA was up-regulated 1300- to 3800-fold 5–11 weeks post co-culture compared to uninfected PBMCs at day 0. As shown in figure 2B PTHrP protein was detectable in the conditioned medium 1 week following co-culture with HTLV-1 producer cells and peak PTHrP protein expression occurred between weeks 10 and 13 post-infection. Peak PTHrP protein expression ranged from 133 to 212 pM in conditioned medium from PBMCs infected with HTLV-1 in the presence of IL-2 and from 130 to 160 pM in conditioned medium from PBMCs infected with HTLV-1 in the absence of IL-2.

### Up-regulation of PTHrP was mediated by the PTHrP P2 and P3 promoters

**Figure 3:**
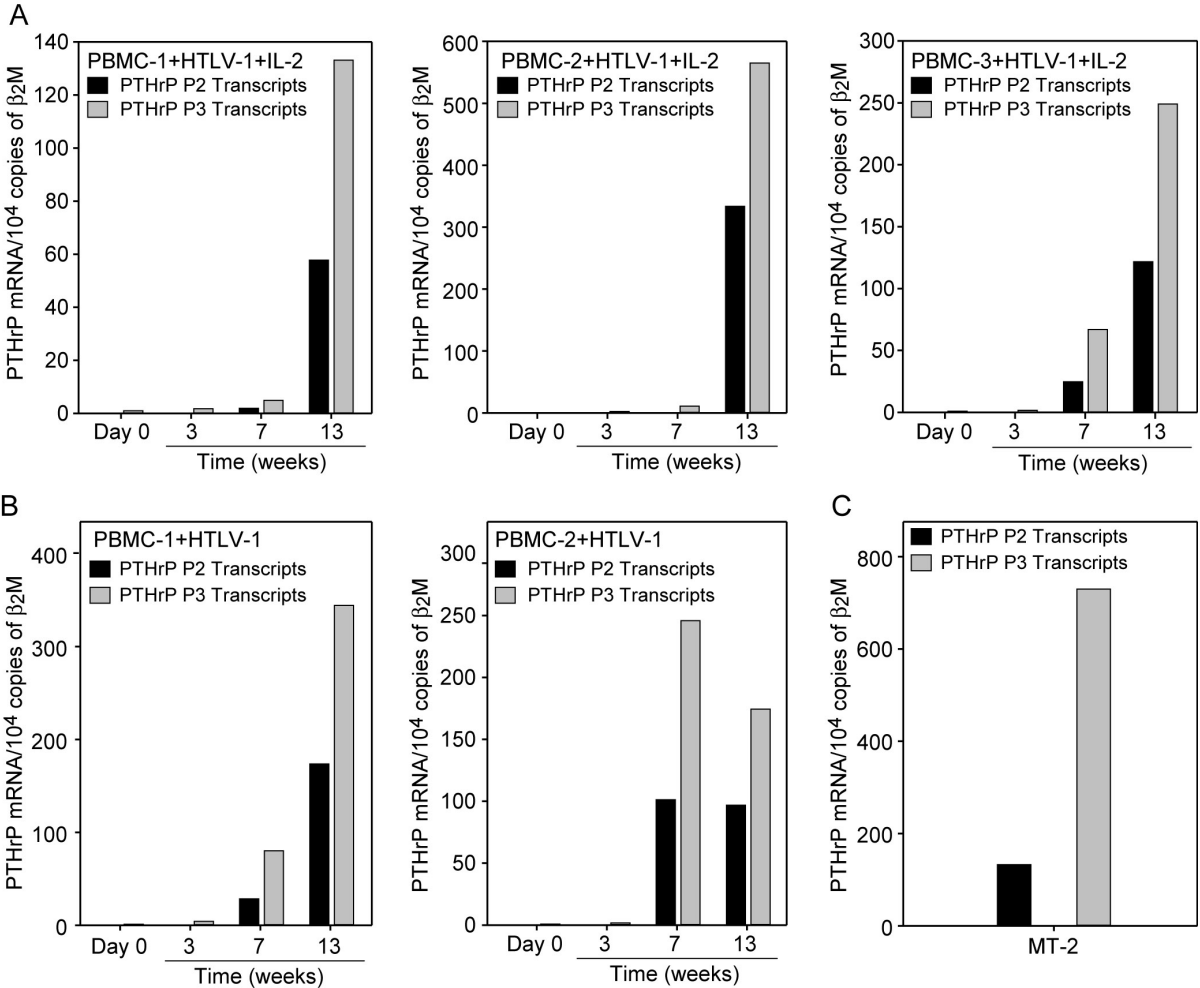
**PTHrP was up-regulated by the P2 and P3 promoters**. Specific PTHrP promoter-initiated transcripts were measured by real-time quantitative RT-PCR using the SYBR green method. The data was normalized to human β_2_M gene expression. Specific PTHrP-promoter initiated transcripts are shown for 0, 3, 7 and 13 weeks post co-culture in the presence of IL-2 (A), in the absence of IL-2 (B) and for MT-2 cells (C). The data showed that PTHrP was up-regulated in PBMCs following HTLV-1 infection by the activation of both the P2 and P3 promoters.

PTHrP is regulated by three distinct promoters that are transactivated by different cellular signal transduction pathways [32]. To understand the molecular mechanisms involved in the transcriptional up-regulation of PTHrP following HTLV-1 infection, we investigated the promoter usage using real-time RT-PCR to detect specific promoter-initiated transcripts. As shown in figure 3, PTHrP P2 and P3 promoters were utilized during immortalization in the presence or absence of IL-2. However, the ratio of P2 to P3 promoter-initiated transcripts was at least 2-fold higher during immortalization of PBMCs with HTLV-1 (1:2) (Figure 3A–B) when compared to transformed MT-2 cells (1:4) (Figure 3C).

### HTLV-1 infection up-regulated PTH1R expression

**Figure 4:**
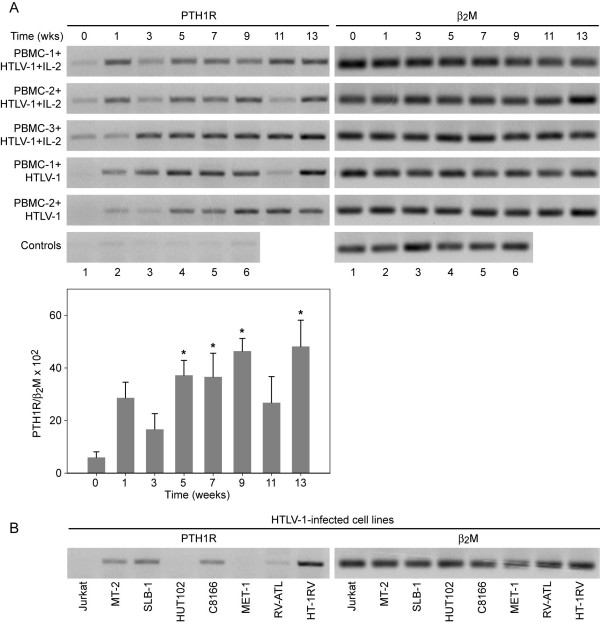
**HTLV-1 infection up-regulated expression of the PTHrP receptor (PTH1R) in PBMCs**. PTHrP receptor expression and human β_2_M were measured by RT-PCR from total RNA at various time points in the co-culture assays. **(A) **Up-regulation of PTH1R in PBMCs at weeks 1, 3, 5, 7, 9, 11, and 13 following HTLV-1 infection in the presence or absence of IL-2 compared to day 0; controls 1 and 4 are PBMC-1 and PBMC-2; controls 2 and 5 are PBMC-1 and PBMC-2 stimulated with PHA; controls 3 and 6 are PBMC-1 and PBMC-2 stimulated with IL-2 for one week. ANOVA with Dunnett's tests were used to analyze the data from PTH1R RT-PCR quantification (bar graph shown at the bottom of the panel). The PTH1R levels were significantly greater at weeks 5, 7, 9, and 13 (p < 0.05; indicated by asterisks in the figure). **(B) **PTH1R expression in HTLV-1-infected T-cells and ATLL cells. Lanes represent: (1) Jurkat (2) MT-2 (3) SLB-1 (4) HUT102 (5) C8166 (6) MET-1 (7) RV-ATL (8) HT-1RV cells. The data showed that PTH1R expression was very low or absent in the ATLL cells (MET-1 and RV-ATL) compared to HTLV-1-infected T-cell lines (MT-2, SLB-1 and HT-1RV). Jurkat T-cells were used as a negative control. β_2_M was used a loading control.

Many of the biological properties of PTHrP result from its interaction with the PTH1R, which is coupled to adenylyl cyclase (AC) and/or phospholipase C (PLC), and downstream signaling pathways [33,34]. Therefore, we measured the expression of PTH1R during immortalization of PBMCs with HTLV-1. As shown in figure 4A, there was very low PTH1R expression in PBMCs. Stimulation of PBMCs with IL-2 or PHA did not up-regulate PTH1R. However, following infection with HTLV-1 there was a marked induction of PTH1R in PBMCs. Singal intensities from the PTH1R were quantitated and averages (PBMC-1, 2, 3 + HTLV-1 + IL-2 and PBMC-1, 2 + HTLV-1 samples combined) were presented as a bar graph in the bottom panel (Figure 4A). The PTH1R levels were significantly greater at weeks 5, 7, 9, and 13 compared to PBMCs alone (p < 0.05). We also analyzed the expression of PTH1R in various HTLV-1-transformed and ATLL cell lines. As shown in figure 4B, HTLV-1-negative Jurkat cells did not express PTH1R. High Tax-expressing HTLV-1-positive cells (MT-2, SLB-1, HT-1RV) expressed moderate levels of PTH1R. RV-ATL cells expressed low levels of PTH1R while MET-1 cells did not express the PTH1R. Human β_2 _microglobulin (B_2_M) was used as a loading control.

### PTHrP expression did not correlate with HTLV-1 tax expression

**Figure 5:**
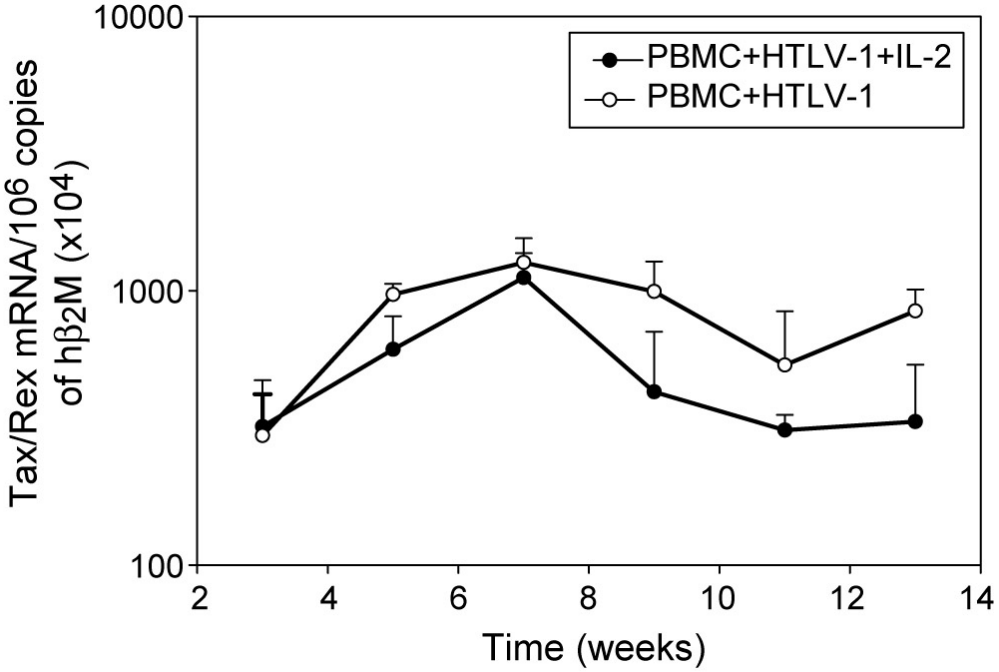
**PTHrP expression did not correlate with HTLV-1 tax expression**. HTLV-1 Tax expression, in co-cultures following HTLV-1 Infection, was measured by quantitative real time RT-PCR using the SYBR green method and the data was normalized to human β_2_M gene expression.

HTLV-1 Tax has been shown to transactivate PTHrP; however, ATLL cells that lack significant Tax expression have very high levels of PTHrP indicating that PTHrP can be expressed in a Tax-independent manner [35]. To investigate the basis for up-regulation of PTHrP due to HTLV-1 infection, we analyzed by quantitative real-time RT-PCR the temporal expression of HTLV-1 viral transcript tax. The high tax expression during the first week in the co-cultures (data not shown) was contributed by the residual live irradiated SLB-1 cells. After the first week, the decline in tax expression correlated with the death of the irradiated SLB-1 cells and the subsequent tax expression was from the newly HTLV-1-infected PBMCs. Tax mRNA expression increased from week 3 to 7 and then decreased between 9–11 weeks post-infection (Figure 5). As shown in figure 5, the expression of tax did not correlate temporally with the expression of PTHrP.

### HTLV-1 and HTLV-1 Rex up-regulated PTHrP expression

**Figure 6:**
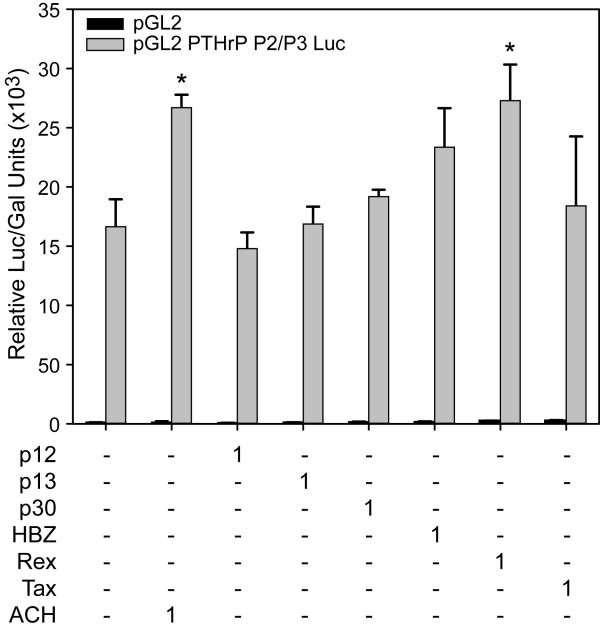
**HTLV-1 infection or over-expression of Rex alone up-regulated PTHrP expression**. Relative luciferase activity in 293T cells transfected with either pGL2 or pGL2 PTHrP-P2/P3 Luc constructs alone or with expression plasmids for HTLV-1 (ACH), p12, p13, p30, HBZ, Rex and Tax. The quantity of the expression plasmid is indicated in μg. Bars represent the mean ± SD of three independent samples. Relative Luc/Gal units were significantly different across groups (p = 0.0002). After adjusting for multiple times of comparison, P2/P3Luc+Rex group and P2/P3Luc+ACH group had significantly greater relative Luc/Gal units than the P2/P3Luc group (p = 0.0006, p = 0.0012, respectively; indicated by asterisks in the figure).

In order to investigate the direct effect of the HTLV-1 viral proteins on PTHrP expression, we co-transfected a PTHrP P2/P3 promoter-driven luciferase plasmid with expression plasmids for HTLV-1 (ACH), p12, p13, p30, Tax, Rex and HBZ (Figure 6). Expression of the HTLV-1 ACH proviral clone or Rex up-regulated PTHrP expression (1.6-fold) 48 h after transfection. The expression of HTLV-1-p12, p13, p30, HBZ or Tax cDNA vectors did not alter PTHrP expression (Figure 6).

### MIP-1α expression correlated with activation of PBMCs following HTLV-1 infection

**Figure 7:**
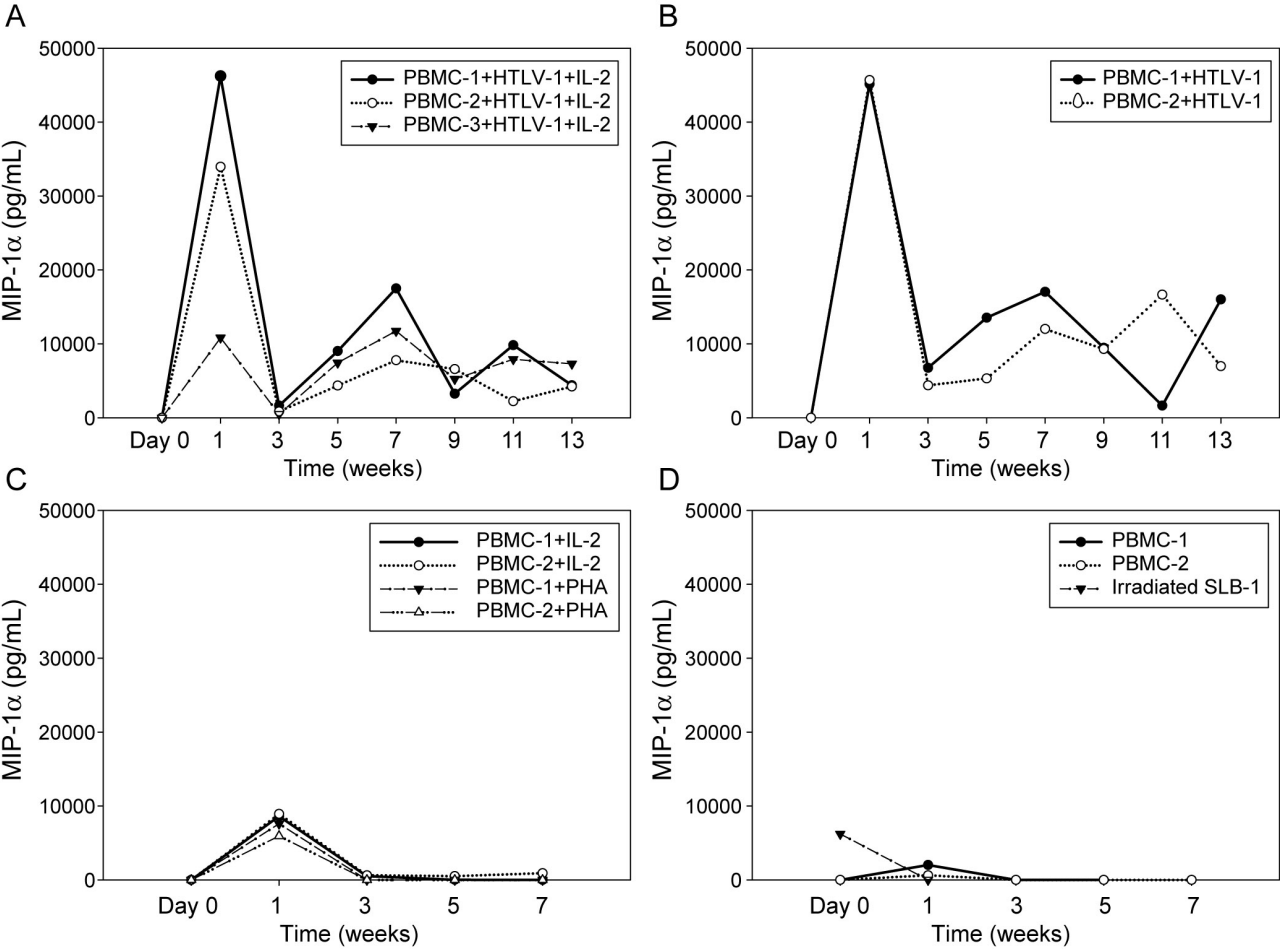
**MIP-1α induction due to activation of lymphocytes following HTLV-1 infection**. MIP-1α was measured in the conditioned medium from the co-culture assays at various time points (day 0, 1, 3, 5, 7, 9, 11 and 13 weeks of co-culture) by ELISA. The results showed that MIP-1α expression was up-regulated in PBMCs following stimulation with PHA or IL-2. HTLV-1 infection markedly up-regulated MIP-1α expression in the first week after infection which demonstrated that HTLV-1 infection activated the lymphocytes. MIP-1α levels were significantly different across groups and time. Overall, MIP-1α levels significantly decreased over time (p < 0.0001). After using Dunnett's method to adjust for multiple comparisons, the HTLV-1-treated groups had significantly higher MIP-1α levels than the PBMC group (p < 0.0001).

Since PTHrP was specifically up-regulated during the immortalization of PBMCs with HTLV-1, we also measured the expression of MIP-1α, another chemokine known to be involved the pathogenesis of HHM in ATLL patients [22]. As shown in figure 7, MIP-1α expression was induced by IL-2 (4- to 14-fold) or PHA (3- to 9-fold) stimulation of PBMCs as expected [36,37], followed by a return to near-baseline concentrations by week 3 (Figure 7C). However, there was marked up-regulation of MIP-1α in the first week post co-culture in PBMCs infected with HTLV-1. The expression of MIP-1α in the immortalization assays ranged from 10,000 to 46,000 pg/mL. After the peak induction of MIP-1α at week 1, there was consistent but lower MIP-1α expression throughout all time points (Figure 7A & B). PBMCs and irradiated SLB-1 cells expressed very low levels of MIP-1α (Figure 7D).

## Discussion

Although HTLV-1 Tax is known to have pleiotropic effects that either directly or indirectly contribute to immortalization and transformation of infected T-cells, the exact mechanisms of transformation are unclear. In this study, we analyzed the temporal PTHrP gene expression during virus-mediated immortalization of lymphocytes to characterize its role in the transformation process. We present data to show that PTHrP is markedly up-regulated during the immortalization process.

An important step in HTLV-1-induced leukemogenesis is the induction of abnormal T-cell growth. Long-term immortalization assays have been used to study the kinetics of HTLV-1 infection and abnormal T-cell growth that lead to transformation. The growth curves in our study are similar to previous reports [31,38]. Human PBMCs that were cultured in the presence of IL-2, but not exposed to the virus, survived *in vitro *only for a few weeks. Following exposure to HTLV-1, PBMCs initially underwent a proliferative response due to HTLV-1 infection after which the cells entered a "growth crisis" between weeks 5–7 followed by expansion of immortalized cells. The high level of HTLV-1 p19 antigen expression in the first few weeks of co-culture was due to the live residual irradiated SLB-1 cells. However, the p19 expression after three weeks in culture was from the newly infected PBMCs and demonstrated active HTLV-1 viral infection (data not shown).

Our data showed that PTHrP mRNA expression was gradually up-regulated in PBMCs following HTLV-1 infection; however, marked expression of PTHrP protein occurred at the time when the PBMCs were undergoing immortalization. This supports an important role for PTHrP during immortalization and the subsequent transformation process. The differences between the levels of PTHrP mRNA and protein expression were likely due to differences in translation efficiency, processing of the mature protein, and/or its secretion from the cells. Regulation of PTHrP secretion is a complex process and it has been shown that some PTHrP may not be secreted but targeted directly to the nucleus and function in an intracrine fashion [39]. Abundant expression of PTH1R is normally found in the target organs that regulate calcium ion homeostasis, such as the kidney and bone, with restricted expression in other tissues. This contrasts with the widespread expression of PTHrP. In our investigation, the marked induction of both PTHrP and PTH1R by HTLV-1 suggests that PTHrP functioned as an autocrine growth regulator in the transformation process.

PTHrP is a complex gene that is regulated by three distinct promoters, P1, P2 and P3, and is transactivated by diverse cellular signal transduction pathways. We and others have shown that the P3 promoter in ATLL cells is regulated by the ETS signaling pathway [45,46] and, recently, we have shown that the P2 promoter is regulated by the NF-κB pathway [47]. Our data in this investigation demonstrated that PTHrP was up-regulated during immortalization through both the P2 and P3 promoters. The ratio of the P2/P3 promoter-initiated transcripts during the immortalization phase was higher (1:2) than in human HTLV-1-transformed T-cells (MT-2; 1:4) or ATLL cells (data not presented) [46]. NF-κB is known to play an important role during the immortalization process and our data showed that the P2 promoter was highly expressed during immortalization. This suggests that NF-κB activity was responsible for transactivating the PTHrP P2 promoter during immortalization.

HTLV-1 tax has been shown to transactivate PTHrP. However, ATLL cells with no significant Tax expression have very high levels of PTHrP. Recently, we have shown that Tax mRNA expression was inversely proportional to PTHrP mRNA expression and PTHrP can be regulated in a Tax-independent manner in ATLL cells [46]. To investigate possible mechanisms for up-regulation of PTHrP in our co-culture assays, we measured the expression of Tax/Rex mRNA. Our data showed that there was no correlation between PTHrP and Tax/Rex mRNA expression. Therefore, induction of PTHrP could either be due to an indirect effect of Tax or possibly a Tax-independent mechanism.

Data from the transfection experiments showed that HTLV-1 infection up-regulated PTHrP expression mildly and suggested that additional cellular events were required to induce the high level PTHrP expression seen in ATLL cells. Alternatively, PTHrP expression might be dependent on cell-type and require lymphocyte-specific factors for marked up-regulation. Over-expression of Rex alone resulted in the up-regulation of PTHrP. Interestingly, Rex and PTHrP have a similar nuclear transport signal and can bind to CRM1 [39,48]. Therefore, the increased expression of PTHrP in the presence of Rex may have been due to increased nuclear export of PTHrP or alternatively due to increased PTHrP mRNA stability since Rex increases the mRNA stability of some genes, such as IL-2Rα [49].

We analyzed the expression of MIP-1α, a second cellular gene that is known to play an important role in the pathogenesis of HHM, in the co-cultures. The data showed that MIP-1α was markedly up-regulated as early as 1 week following HTLV-1 infection of PBMCs. These data are in agreement with reports that showed MIP-1α was up-regulated during activation of T-lymphocytes [50]. Our data demonstrated that MIP-1α was up-regulated early in the co-cultures with HTLV-1 infection due to activation of T-lymphocytes. In contrast, up-regulation of PTHrP occurred later during the immortalization, which supported a specific role for PTHrP in the transformation process.

## Conclusion

Our data demonstrated that PTHrP was dramatically and specifically up-regulated during the immortalization of PBMCs with HTLV-1 in a Tax-independent manner. PTHrP likely functioned in an autocrine manner with the PTH1R facilitating the transformation process. Although further investigations are required to understand the role of PTHrP in the transformation process, it is apparent that PTHrP is up-regulated not only during HHM but also during early HTLV-1 infection implicating an important dual role for PTHrP in the pathogenesis of ATLL. Novel therapies directed against PTHrP will be an important strategy to prevent ATLL in HTLV-1-infected patients.

## Materials and methods

### Cells

293T cells were maintained in Dulbecco's modified eagle medium (DMEM) supplemented with 10% fetal bovine serum (FBS), 2 mM glutamine, penicillin (100 U/mL), and streptomycin (100 μg/mL). PBMCs were cultured in RPMI 1640 medium supplemented with 20% FBS, 2 mM glutamine, and antibiotics in the presence or absence of 10 U/mL IL-2 (Boehringer Mannheim, Mannheim, Germany).

### Long-term co-culture assays

PBMCs were isolated from the blood of healthy donors by centrifugation over Ficoll-Paque (Pharmacia, Piscataway, NJ). Long term co-culture assays were performed as described previously [51]. Briefly, 2 × 10^6 ^PBMCs were cultured alone or co-cultured with 10^6 ^SLB-1 producer cells (in approximately 2 mL of culture medium) irradiated with 10,000 rad in 24-well culture plates in the absence (PBMC-1, 2 + HTLV-1; PBMC-1, 2 represent PBMCs from two different donors) or presence of 10 U/mL human IL-2 (hIL-2) (PBMC-1, 2, 3 + HTLV-1+ IL-2; PBMC-1, 2, 3 represent PBMCs from three different donors). Viable cells were counted weekly by trypan blue exclusion. Cells that continued to produce p19 Gag antigen and proliferate 12 weeks after co-culture were identified as HTLV-1-immortalized. PBMCs cultured alone (PBMC-1, PBMC-2) or the in the presence of IL-2 (PBMC-1 + IL-2, PBMC-2 + IL-2) or phytohemagglutinin (PHA) (PBMC-1+PHA, PBMC-2 + PHA) without HTLV-1 infection were used as controls.

### Real time RT-PCR

Total RNA was extracted using TRIZOL^® ^Reagent (Invitrogen, Carlsbad, CA). To measure the total PTHrP mRNA, 1 μg RNA was reverse-transcribed and amplified by real-time RT-PCR analysis using TaqMan^® ^Gene Expression assays (4331182, Applied Biosystems, CA). β2M (4333766, Applied Biosystems) was used as a reference gene. PTHrP P2 and P3 promoter-initiated transcripts, PTH1R and HTLV-1 Tax mRNAs were measured as described previously [38,52,53]. The PTH1R gels were scanned with a Typhoon 9410 Variable Mode Imager (GE Healthcare Bio-Sciences Corp.) and PTH1R PCR products were quantified using ImageQuant TL Version 7.0 software.

### PTHrP Immunoradiometric Assay

PTHrP concentrations were measured in the conditioned medium using a two-site immunoradiometric assay (DSL, Webster, TX) specific for the PTHrP N-terminal region (amino acids 1 to 40) and mid-region (amino acids 57 to 80).

### Enzyme Linked Immunosorbant Assays

p19 Gag protein in the culture supernatant was measured using a commercially available ELISA kit (Zeptometrix, Buffalo, NY). MIP-1α protein in the conditioned medium was measured using the Quantikine Human CCL3/MIP-1α Immunoassay (R&D systems, Minneapolis, MN).

### Plasmids and transfections

The PTHrP P2/P3 luciferase construct was made by cloning the PTHrP P2/P3 promoter fragment (-1120 Bam H1 to +1 Hind III) into the pGL2 basic vector. ACH, pcTax, BCRex, HBZ plasmids were obtained from the laboratory of Dr. Patrick Green (The Ohio State University). p12, p13 and p30 expression plasmids were obtained from laboratory of Dr. Michael Lairmore (The Ohio State University). 293T cells were transfected with either PTHrP P2/P3 PGL2 Luc plasmid alone or with ACH, pcTax, BCRex, HBZ, p12, p13, p30 vectors. pcDNA-3.1 was used as a "filler" plasmid so that the total amount of DNA would be the same in all transfection groups. The plasmid pβgal-Control Vector (250 ng) was included in each transfection and served as an internal control to correct for transfection efficiency. Luciferase activity was measured with the Luciferase Assay System (Promega) using 40 μl of lysate. Simultaneously, β-galactosidase activity was measured with the Luminescent β-Galactosidase Detection Kit II (BD Biosciences).

### Statistical analyses

For the co-culture experiments, linear mixed models with repeated measures (ANOVA with repeated measures) were used to study the effects of time, treatment and the interaction between time and treatment. The square-root transformation was used for cell number and MIP-1α data to achieve normality and homogeneous variances. Dunnett's method was used to adjust for multiple comparisons versus the control group. In some treatments (PBMC, PBMC+IL2, PBMC+PHA), cell numbers and protein level were zero after 6 weeks. Thus, a non-parametric method (Wilcoxon sum rank) was used for the comparison among non-zero groups to the zero groups after week 6. ANOVA with Dunnett's tests were used to analyze the data from transfection experiments and PTH1R RT-PCR quantification. A multiplicity-adjusted p value less than alpha = 0.05 was considered significant. In the figures, either raw data or averages were plotted to improve readability and visualization of the data.

## Competing interests

The authors declare that they have no competing interests.

## Authors' contributions

MVPN, SS, WPD, NKT, NKS, ML, PLG, SAF, MDL and TJR have all met the definition of author as outlined by the *Retrovirology *journal.
